# Supplementary material for: Downregulation of miR-182-5p by NFIB promotes NAD+ salvage synthesis in colorectal cancer by targeting NAMPT
Source: Commun Biol. 2023 Jul 25;6:775. doi: 10.1038/s42003-023-05143-z (PMC10368701; doi:10.1038/s42003-023-05143-z)
Supplement: Supplementary file 2 — Supplementary materials [file 42003_2023_5143_MOESM2_ESM.pdf]

Downregulation of miR-182-5p by NFIB promotes NAD+ salvage synthesis in colorectal cancer by targeting NAMPT

Li Zhou[1], Hongtao Liu[1], Zhiji Chen[1], Siyuan Chen[1], Junyu Lu[1], Cao Liu[2], Siqi Liao[1], Song He[1], Shu Chen[3*], Zhihang Zhou[1*]

[1] Department of Gastroenterology, The Second Affiliated Hospital of Chongqing Medical University, Chongqing, 400010, China.

[2] Department of Emergency, The General Hospital of Xinjiang Military Command, Urumqi, 830000, China.

[3] Department of Hematology, The Second Affiliated Hospital of Chongqing Medical University, Chongqing, 400010, China.

*Correspondence: Zhihang Zhou, zhouzhihang@cqmu.edu.cn; Shu Chen, chenshu921@163.com.

Supplementary Figures

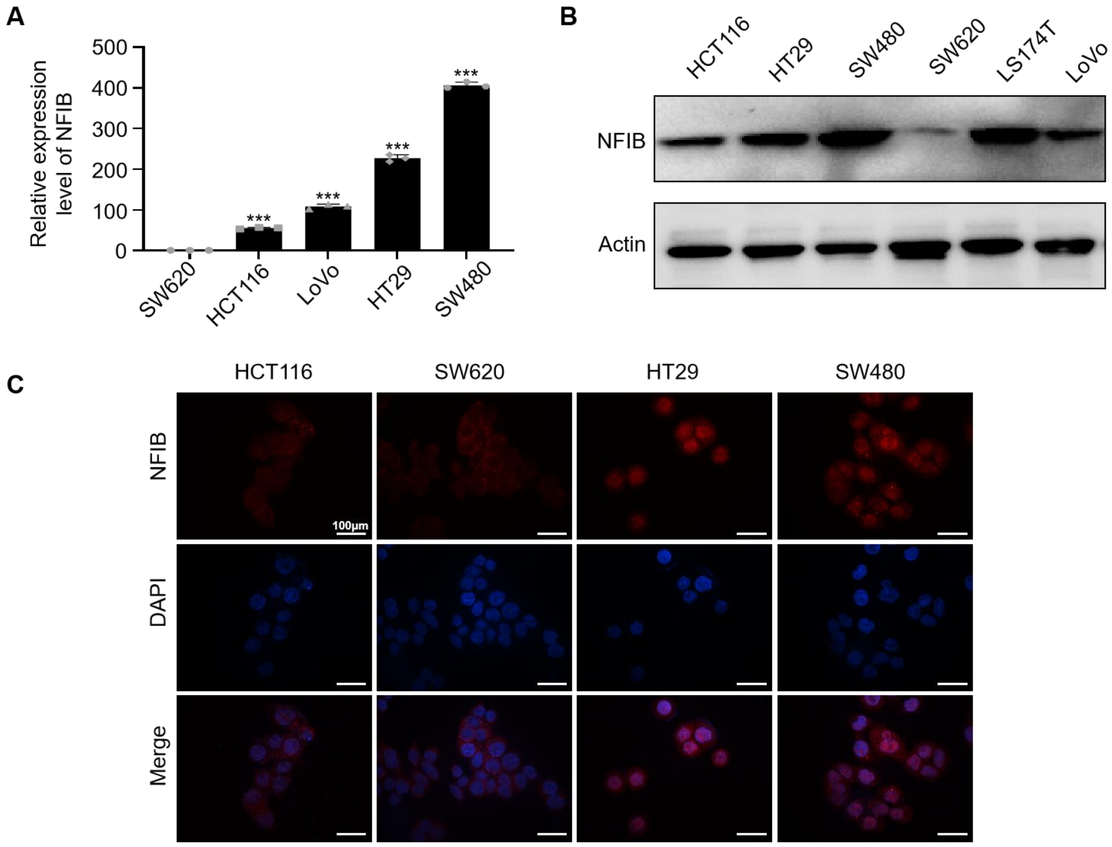

Supplementary Figure 1. Expression of NFIB in wild-type CRC cell lines. A and B Expression levels of NFIB in wild-type CRC cell lines. **C** Immunofluorescence expression of NFIB in HCT116, SW620, HT29 and SW480 cells. Data are presented as means ± SD of three independent experiments. Student's t-test was used for statistical analysis. ***P<0.001.

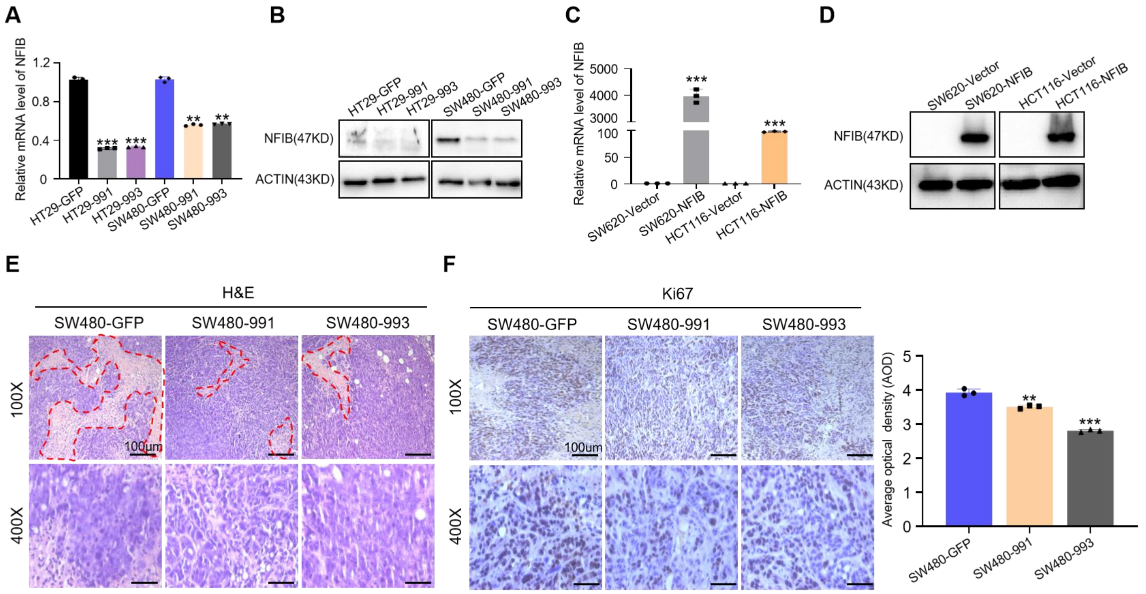

Supplementary Figure 2. knockdown or overexpression NFIB in CRC cells. A-D qRT-PCR and western blot were used to verify the efficiency of HT29 and SW480 knockdown of NFIB (A and B) and SW620, HCT116 overexpression of NFIB (C and D). **E and F** H&E and Ki67 staining of mouse xenografts showed that the malignancy of the tumor cells are reduced after NFIB knockdown. Data are presented as means ± SD of at least three independent experiments. Student's t-test was used for statistical analysis. Scale bar: 100μm. GFP and Vector represent control group, 991 and 993 represent NFIB-knockdown group, NFIB represents NFIB overexpression group. **P<0.01; ***P<0.001.

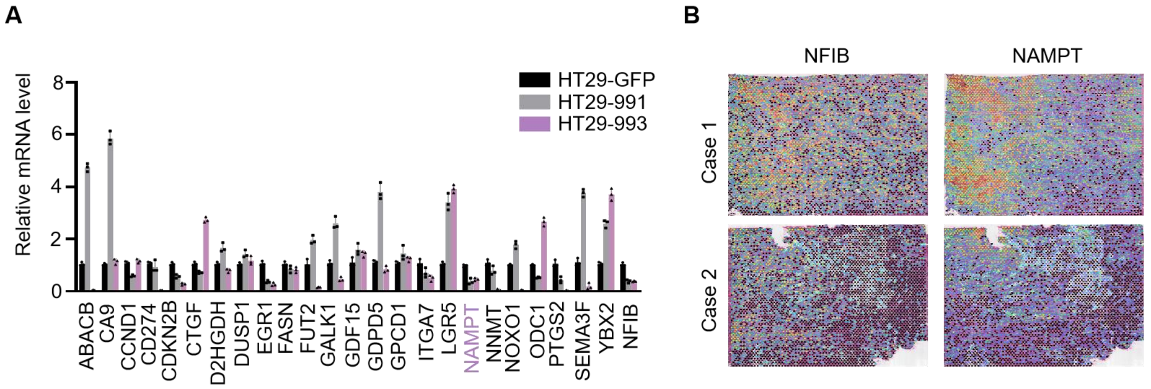

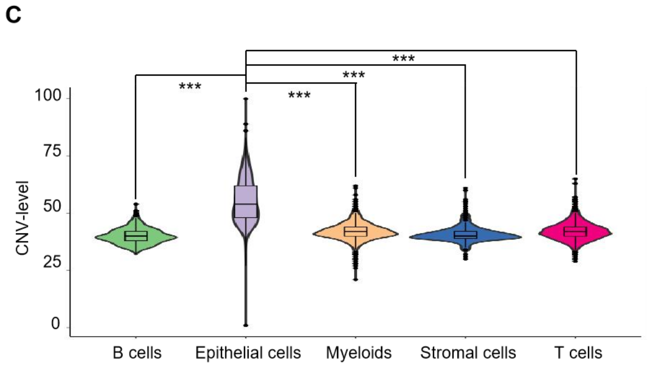

D

Name	Fold change	P value
hsa-miR-182-5p	2.08	0
hsa-miR-26b-5p	2.09	0
hsa-miR-26a-5p	2.21	0
hsa-miR-34a-5p	2.36	<0.001
hsa-miR-374a-3p	2.44	<0.001
hsa-miR-374a-5p	3.01	0
hsa-miR-206	4.49	<0.001

Supplementary Figure 3. The relationship between NFIB and NAMPT. A qRT-PCR was used to verify the expression of related genes in HT29 cells after NFIB knockdown. **B** Whole Transcriptomic analysis showed that the expression regions of NFIB and NAMPT partially overlapped in the cancer tissues of the two colon cancer patients (case 1 and case 2), as shown in the orange spot. **C** Violin diagram showed that CNV levels were significantly higher in epithelial cells than in other cell subgroups ($p<0.001$). **D** Top miRNA up-regulated after NFIB knockdown. ***$P<0.001$.

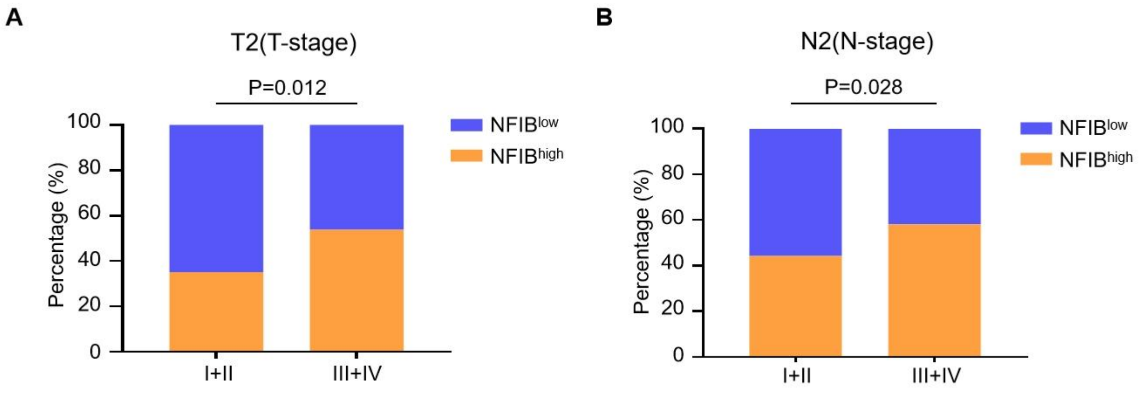

Supplementary Figure 4. The relationship between NFIB and clinical outcome. A and B Chi-square test showed the relationship between NFIB expression and T-stage (A), N-stage (B) in CRC.

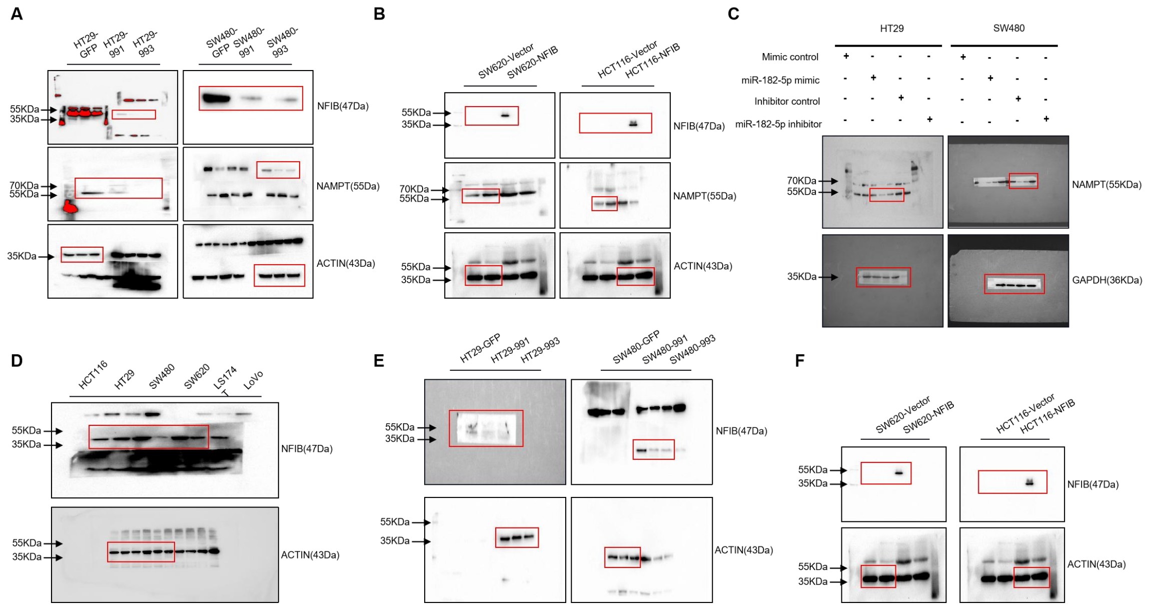

Supplementary Figure 5. The Uncropped and unedited blotgel images in figures. A Uncropped and unedited blotgel images in figure 3c. **B** Uncropped and unedited blotgel images in figure 3e. **C** Uncropped and unedited blotgel images in figure 4f. **D** Uncropped and unedited blotgel images in Supplementary Figure 1b. **E** Uncropped and unedited blotgel images in Supplementary Figure 2b. **F** Uncropped and unedited blotgel images in Supplementary Figure 2d.

Supplementary Tables

Supplementary Table 1. The top 20 transcription factors that bind to NAMPT after intersection of UCSC, PROMO and GeneCards databases.

**Gene ID**	**JASPAR(Matrix ID)**	**Data Type**	**Score**	**Relative score**
ZNF460	MA1596.1	ChIP-seq	23.795557	0.96483588
ZNF135	MA1587.1	ChIP-seq	17.904411	0.935251465
MYBL2	MA0777.1	HT-SELEX	16.195566	0.912770221
MEF2C	MA0497.1	ChIP-seq	15.601086	0.943150985
SATB1	MA1963.1	ChIP-seq	15.115449	0.966691961
STAT3	MA0144.2	ChIP-seq	14.70235	0.975667142
FOXF2	MA0030.1	SELEX	13.754334	0.913254809
POU5F1	MA1115.1	ChIP-seq	13.605302	0.968595578
SREBF2	MA0596.1	ChIP-seq	13.58836	0.959722822
MEF2A	MA0052.1	SELEX	12.906454	0.913583225
MEF2D	MA0773.1	HT-SELEX	12.632074	0.917390414
Hoxa13	MA0650.1	HT-SELEX	12.581893	0.94289916
Msgn1	MA1524.1	HT-SELEX	11.453627	0.948781364
USF2	MA0526.1	ChIP-seq	11.013265	0.92309021
FOXD2	MA0847.1	HT-SELEX	10.600332	0.977826697
FOXP3	MA0850.1	HT-SELEX	10.445832	0.978500055
SP1	MA0079.1	SELEX	9.304687	0.911425279
SPI1	MA0080.1	SELEX	8.578765	0.994463805
YY1	MA0095.1	COMPILED	8.38313	0.999999988
c-Ets-1	MA0098.1	SELEX	7.632889	0.992421538

Supplementary Table 2. The primers used in the study.

**Gene**	**Forward**	**Reverse**
NFIB	CTGGAAGTCGAACATGGC	GGGAAGAATCCTGTGGAGA
NAMPT	TATTGAACTGGAAGCAGCAC	AAAACACAAACCCCACACA
ACACB	CATGGCAAGAGAAAAGCGGC	ACTCTTGGTGATCGGCTTGG
CA9	CTTCTGGTGCCTGTCCATC	GATCCTCCTCGCCCAGT
CCND1	AGAGGCGGAGGAGAACA	GAGAGGAAGCGTGTGAGG
CD274	GCGAAAGCAGAGGAGGA	TCACAGGCGTCGATGAG
CDKN2B	TGCAAGCGACGACAGAT	CACTCCACCACCTCATCC
CTGF	GAAATGCTGCGAGGAGTG	CCCACAGGTCTTGGAACA
D2HGDH	GGCAGGCACCCTCCTTT	GAGACCCACCCCGCTTC
DUSP1	CGGAATCTGGGTGCAGTT	TGCGGGAAGCGTGATAC
EGR1	TGCTAAAGGGAAAGGGGAA	TTGGGGAAGGGGAAGTG
FASN	CGAGCTGCACCATCATCC	AAGGCATTGGGGTTGGC
FUT2	GCCCATTCTTTGAGACCTT	CCCAACGCATCTTCACA
GALK1	TCTGAGGGTGCCGATGAG	TGCACTGAAGCCAGGGA
GDF15	GGTGTTGCTGGTGCTCTCGTG	TCGGAATCTGGAGTCTTCGGAGTG
GDPD5	CTCACGGGCATCTACGG	CCCACCAGAAGTAAAGCCA
GPCPD1	AACCCGGAGCACGACATCAT	CCAAAGCATCACAGCTTCCAC
ITGA7	GGAAGAACCCAAGCACCA	GTCACTACAATGGCCCGAA
LGR5	CAGGATGTTGCTCAGGGT	AGGTTGGAAGGCAGCTC
NNMT	TCAGCCAACATTCCTTAGC	GCACCATCATTCTTCTCGT
NOXO1	GTTTCCAGCGCCCTACC	GGCACGGACAGCTCATC
ODC1	TGGGTGATTGGATGCTCT	GAAGTCGGGGTTCTGGA
PTGS2	GAGCACCATTCTCCTTGAAA	ATTGAGGCAGTGTTGATGATT
SEMA3F	CTTGTCGCCGGTCTTCTT	TCGGTAGTCGGTTGTGTTG
YBX2	CCCACCCTTCTTCTACCG	TCATCTCCCTGCTGTTGG

RNU6	CTCGCTTCGGCAGCACA	AACGCTTCACGAATTTGCGT
GAPDH	GTATGACAACGAATTTGGCTACAG	TGAGGGTCTCTCTCTTCCTCTTGT
hsa-miR-182-5p	ccgcgTTTGGCAATGGTAGAACTCACACT	
hsa-miR-26b-5p	cgcgcgcgTTCAAGTAATTCAGGATAGGT	
hsa-miR-26a-5p	ccggcgTTCAAGTAATCCAGGATAGGCT	
hsa-miR-374a-3p	gcgcgccgcgCTTATCAGATTGTATTGTAATT	
hsa-miR-374a-5p	gcgTGGCAGTGTCTTAGCTGGTTGT	
hsa-miR-206	gcgcgTGGAATGTAAGGAAGTGTGTGG	
