# Supplementary material for: Downregulation of miR-182-5p by NFIB promotes NAD+ salvage synthesis in colorectal cancer by targeting NAMPT
Source: Commun Biol. 2023 Jul 25;6:775. doi: 10.1038/s42003-023-05143-z (PMC10368701; doi:10.1038/s42003-023-05143-z)
Supplement: Supplementary file 3 — Reporting Summary [file 42003_2023_5143_MOESM3_ESM.pdf]

nature portfolio

Corresponding author(s): COMMSBIO-23-0075B

Last updated by author(s): Jun 29, 2023

Reporting Summary

Nature Portfolio wishes to improve the reproducibility of the work that we publish. This form provides structure for consistency and transparency in reporting. For further information on Nature Portfolio policies, see our Editorial Policies and the Editorial Policy Checklist.

Statistics

For all statistical analyses, confirm that the following items are present in the figure legend, table legend, main text, or Methods section.

n/a	Confirmed	
☐	☒	The exact sample size (*n*) for each experimental group/condition, given as a discrete number and unit of measurement
☐	☒	A statement on whether measurements were taken from distinct samples or whether the same sample was measured repeatedly
☐	☒	The statistical test(s) used AND whether they are one- or two-sided<br>*Only common tests should be described solely by name; describe more complex techniques in the Methods section.*
☒	☐	A description of all covariates tested
☒	☐	A description of any assumptions or corrections, such as tests of normality and adjustment for multiple comparisons
☒	☐	A full description of the statistical parameters including central tendency (e.g. means) or other basic estimates (e.g. regression coefficient) AND variation (e.g. standard deviation) or associated estimates of uncertainty (e.g. confidence intervals)
☒	☐	For null hypothesis testing, the test statistic (e.g. *F*, *t*, *r*) with confidence intervals, effect sizes, degrees of freedom and *P* value noted<br>*Give P values as exact values whenever suitable.*
☒	☐	For Bayesian analysis, information on the choice of priors and Markov chain Monte Carlo settings
☒	☐	For hierarchical and complex designs, identification of the appropriate level for tests and full reporting of outcomes
☒	☐	Estimates of effect sizes (e.g. Cohen's *d*, Pearson's *r*), indicating how they were calculated

Our web collection on statistics for biologists contains articles on many of the points above.

Software and code

Policy information about availability of computer code

Data collection	No software was used.
Data analysis	Graphpad prism 8.0.

For manuscripts utilizing custom algorithms or software that are central to the research but not yet described in published literature, software must be made available to editors and reviewers. We strongly encourage code deposition in a community repository (e.g. GitHub). See the Nature Portfolio guidelines for submitting code & software for further information.

Data

Policy information about availability of data

All manuscripts must include a data availability statement. This statement should provide the following information, where applicable:

- Accession codes, unique identifiers, or web links for publicly available datasets
- A description of any restrictions on data availability
- For clinical datasets or third party data, please ensure that the statement adheres to our policy

The datasets generated during and/or analysed during the current study are available from the corresponding author on reasonable request

Research involving human participants, their data, or biological material

Policy information about studies with human participants or human data. See also policy information about sex, gender (identity/presentation), and sexual orientation and race, ethnicity and racism.

Reporting on sex and gender	n/a
Reporting on race, ethnicity, or other socially relevant groupings	n/a
Population characteristics	n/a
Recruitment	n/a
Ethics oversight	n/a

Note that full information on the approval of the study protocol must also be provided in the manuscript.

Field-specific reporting

Please select the one below that is the best fit for your research. If you are not sure, read the appropriate sections before making your selection.

☒ Life sciences ☐ Behavioural & social sciences ☐ Ecological, evolutionary & environmental sciences

For a reference copy of the document with all sections, see nature.com/documents/nr-reporting-summary-flat.pdf

Life sciences study design

All studies must disclose on these points even when the disclosure is negative.

Sample size	From November 2012 to 2015,261 pairs of CRC tissues and adjacent normal mucosa samples were collected from the hospital.
Data exclusions	Patients were excluded if they received radiotherapy or chemotherapy before surgery, presented with other malignant diseases within the past 5 years,were lost to follow-up,or exhibited incomplete clinicopathological data.
Replication	All experimental data were repeated three times.
Randomization	This study did not involve a randomized trial.
Blinding	This study did not involve a blind study.

Reporting for specific materials, systems and methods

We require information from authors about some types of materials, experimental systems and methods used in many studies. Here, indicate whether each material, system or method listed is relevant to your study. If you are not sure if a list item applies to your research, read the appropriate section before selecting a response.

Materials & experimental systems

n/a	Involved in the study	
☐	☒	Antibodies
☐	☒	Eukaryotic cell lines
☒	☐	Palaeontology and archaeology
☐	☒	Animals and other organisms
☐	☒	Clinical data
☒	☐	Dual use research of concern
☒	☐	Plants

Methods

n/a	Involved in the study	
☐	☒	ChIP-seq
☒	☐	Flow cytometry
☒	☐	MRI-based neuroimaging

Antibodies

Antibodies used	NFIB (Abcam,UK,1:1000 dilution), NAMPT (Abcam,UK,1:1000 dilution), ACTIN (Proteinates,China), and GAPDH (Proteinates,China).
Validation	All antibodies are available on the supplier's website.

Eukaryotic cell lines

Policy information about cell lines and Sex and Gender in Research

Cell line source(s)	Human CRC cell lines (HT29, HCT-116, SW620, SW480, LoVo, and LS174T) were purchased from the Culture Collection of the Chinese Academy of Sciences (Shanghai,China).
Authentication	None of the cell lines used were authenticated.
Mycoplasma contamination	All cell lines tested negative for mycoplasma contamination.
Commonly misidentified lines (See ICLAC register)	n/a

Animals and other research organisms

Policy information about studies involving animals; ARRIVE guidelines recommended for reporting animal research, and Sex and Gender in Research

Laboratory animals	BALB/c athymic male nude mice (4 weeks old) were obtained from Charles River Biotechnology (Beijing, China).
Wild animals	The study did not involve wild animal.
Reporting on sex	Only male mice were used in this study because healthy male mice were selected to reduce the difference in tumor formation caused by sex.
Field-collected samples	All mice were fed and observed under laboratory standard conditions.
Ethics oversight	All animal experiments were approvedby the Animal Care and Use Committee.

Note that full information on the approval of the study protocol must also be provided in the manuscript.

Clinical data

Policy information about clinical studies

All manuscripts should comply with the ICMJE guidelines for publication of clinical research and a completed CONSORT checklist must be included with all submissions.

Clinical trial registration	n/a
Study protocol	The expression difference of NFIB in clinical patients with CRC and para-cancer was compared, and whether there was a correlation with the depth and stage of invasion was statistically analyzed.
Data collection	All patients met the following inclusion criteria: underwent enterectomy and were confirmed by pathological diagnosis; complete clinicopathological data, including gender, age, tumor size, tumor differentiation, pT stage, pN stage, distant metastasis, pTNM, vascular invasion, and nerve invasion; complete follow-up information; and written informed consent.
Outcomes	Death and recurrence.

Plants

Seed stocks	n/a
Novel plant genotypes	n/a
Authentication	n/a

ChIP-seq

Data deposition

☒ Confirm that both raw and final processed data have been deposited in a public database such as GEO.

☒ Confirm that you have deposited or provided access to graph files (e.g. BED files) for the called peaks.

Data access links *May remain private before publication.*	https://www.ncbi.nlm.nih.gov/geo/query/acc.cgi?acc=GSE235617
Files in database submission	ChIP-seq

Genome browser session (e.g. UCSC)	no longer applicable.

Methodology

Replicates	Three biological samples were repeated.
Sequencing depth	The sequencing depth was 20-40M,they were paired-end.
Antibodies	Anti-NFIB or rabbit anti-IgG antibodies were used for precipitation.
Peak calling parameters	Peaking calling was performed based on the filtered sequence data.
Data quality	Peaks were called using the MACS2 algorithm and SICER at FDR < 1%.
Software	Hiseq 2500 platform (Ilumina, USA).
